# Recent Advances in Biological and Technological Research of Fresh Fruit and Vegetable

**DOI:** 10.3390/foods13132092

**Published:** 2024-07-01

**Authors:** Zhongqi Fan

**Affiliations:** College of Food Science, Fujian Agriculture and Forestry University, Fuzhou 350002, China; fanzqi@fafu.edu.cn

Fresh fruit and vegetables are sources of vitamins, minerals, and dietary fiber; however, due to their short postharvest life, a large portion of the produce is lost. It is, therefore, necessary to investigate the biological changes underlying ripening and senescence and to find appropriate ways to control the perishability of fresh fruit and vegetables, thereby increasing their shelf life [1,2]. The basic science of the ripening and senescence of fruit and vegetables has progressed rapidly in recent years. This is largely due to a series of breakthrough discoveries regarding key factors and signaling pathways that activate ripening-related and senescence-associated genes [3,4]. On the other hand, advanced postharvest technologies, including chemical treatment, physical methods, and biotechnology, have been widely applied to minimize postharvest loss and maintain the commercial quality of fresh fruit and vegetables [5,6]. This Special Issue, “Recent Advances in Biological and Technological Research of Fresh Fruit and Vegetable”, features 15 articles. These articles represent cutting-edge research on the biological mechanisms, physiology disorders, and preservation technologies of fresh fruit and vegetables.

Proteomics and metabolomics technologies are important omics approaches to detect the nutrients in fruit and vegetables [7]. In this Special Issue, an integrating metabolomics and proteomics approach was utilized to investigate the flavor precursor in the maturity stage of *coffea arabica* L. cherries. A total of 456 differently expressed metabolites (DEMs) and 402 differently expressed proteins (DEPs) were identified. Among them, 45 DEPs were revealed to modulate 40 flavor precursors of primary amino acids. The results reveal the amino acid and organic acid metabolic pathways are important for flavor formation during the ripening of coffee cherries [contribution 1]. 

Additionally, HPLC-MS was widely used to determine the chemical substances in various food materials [8]. Regarding the quality of apple juice, HPLC-MS was used to detect changes in phenolic compounds, organic acids, and sugar content after processing and storage. A total of 26 phenolic compounds were identified and quantified in both of red-fleshed cultivar ‘Baya Marisa’ and white-fleshed cultivar ‘Golden Delicious’. The results suggest that phenolic compound preservation of apple juice during storage depends on the fruit cultivar, and refrigeration is the best storage condition for apple juice [contribution 2]. 

Quality deterioration in fresh fruit and vegetables occurs due to the vigorous respiratory metabolism postharvest. Advanced postharvest technologies, including chemical treatment, physical methods, and biotechnology, have been utilized to control product loss and maintain the commercial quality of fresh fruit and vegetables. For physical treatment, a cooling method was used to delay core browning in postharvest ‘Yali’ pear fruit. Slow cooling and rapid cooling treatments were performed to store the pears. The respiration intensity and ethylene production in slow-cooled fruits were higher than that of rapid-cooled fruit. The rapid cooling group exhibited better fruit quality than the slow cooling group. Furthermore, RNA-seq analysis reveal pear browning was associated with hormone signal transduction pathways, especially ethylene metabolism. The results suggest that rapid cooling inhibits core browning in postharvest ‘Yali’ pear by partly regulating ethylene biosynthesis and signal transduction [contribution 3]. 

Controlled atmosphere (CA) packaging has been widely used to prolong the shelf-life of fresh produce. A gas environment with O_2_ (5%), CO_2_ (5%), and N_2_ (90%) was used to treat fresh-cut *Dictyophora rubrovolvata*. This CA treatment protected membrane integrity, prevented oxidation, and inhibited the browning of fresh-cut *Dictyophora rubrovolvata* during 8 days storage at 4 ± 1 °C. Moreover, the quality, taste, and volatile components of fresh-cut *Dictyophora rubrovolvata* were maintained by the CA treatment [contribution 4].

Regarding chemical treatment, ε-poly-L-lysine (ε-PL) combined with a chitosan coating effectively inhibits diseases development and weight loss in fresh *Tremella fuciformis* during storage. ε-PL is a homopolymer composed of lysine residues; it is non-toxic and harmless to humans, displaying efficient endotoxin removal capabilities. Chitosan is a high-molecular-weight cationic polysaccharide that is safe and non-toxic, soluble in various organic acids, and has certain antibacterial, antioxidant, and film-forming adsorption properties. This chemical treatment (ε-PL + chitosan) suppressed the respiration rate and activities of respiratory-metabolism-related enzymes and energy-metabolism-related enzymes. This work demonstrated that the application of ε-PL and chitosan coating has the potential to maintain the quality of postharvest, fresh *Tremella fuciformis* [contribution 5]. 

Another edible coating technology, comprising carboxymethyl cellulose (CMC) and gelatin (Gel) integrated with melatonin (MT), mitigated pericarp browning and chilling injury in longkong fruit. The results showed that the CMC-Gel-MT coating alleviated chilling injury by reducing the activities of cellular-degrading enzymes and improving antioxidant ability [contribution 6]. 

Furthermore, pullulan coating (a bio-polysaccharide produced by *Aureobasidium pullulans*) combined with potassium metabisulfite could reduce the decay rate of kiwifruit and maintain fruit quality during storage, as evidenced by higher levels of soluble solid content and firmness, increased accumulation of phenols and flavonoids, as well as higher activities and genes expression of antioxidant enzymes. Overall, this study suggested that the use of pullulan-based active coating combining with potassium metabisulfite treatment inhibited soft rot and maintained the quality of postharvest kiwifruit [contribution 7]. 

Another study revealed that exogenous triterpenoids, including oleanolic acid (OA) and ursolic acid (UA), effectively reduced water loss and improved the quality of blueberry fruit during storage [contribution 8]. 

Moreover, calcium chloride (CaCl_2_) treatment maintained litchi fruit quality by preventing the activities of cell-wall-degrading enzymes, thereby stabilizing the cell wall’s architecture [contribution 9]. 

Melatonin treatment prolonged postharvest quality and controlled disease development in strawberry fruit by enhancing the reactive oxygen species (ROS) scavenging system [contribution 10]. 

Dry walnut and hazelnut leaves used for composting could affect the yield and phenolic composition of *Lactuca sativa* L. This study suggested that dry walnut leaves and cut grass could be used for composting; however, the dry hazelnut leaves still contained some unknown allelochemicals after two years, which inhibited plant growth and, thus, yield. Therefore, dry hazelnut leaves should not be recommended for composting [contribution 11].

More interestingly, a combined chemical–physical method was explored. Near-freezing temperature (NFT) with jujube polysaccharide treatment could maintain the fruit quality of *Prunus armeniaca* L. Further proteomic analysis identified 1054 differentially expressed proteins (DEPs), which were enriched in carbohydrate and energy metabolism, cell wall degradation, stress response, and antioxidant system [contribution 12]. 

Regarding biocontrol treatment technologies, fruit-isolated *Metschnikowia* yeasts were effective against three major citrus pathogens, including *Penicillium digitatum*, *Penicillium italicum*, and *Geotrichum citri-aurantii*; further investigations revealed that the *Metschnikowia* yeasts’ biocontrol efficacy was mainly attributable to their ability to competitively consume iron ions in a shared environment. Moreover, the size of their pigment halo was directly related to their antagonistic ability [contribution 13]. 

Additionally, the antifungal effects of the volatile organic compounds (VOCs) produced by *Scheffersomyces spartinae* W9 (a marine biocontrol yeast) were evaluated against *Botrytis cinerea* on strawberry fruit. The findings displayed a biocontrol strategy to prevent disease development in strawberry fruit [contribution 14].

Regarding molecular studies, a cold-induced WRKY transcription factor, MaWRKY70, was associated with banana chilling injury development by directly binding to the promoters of four lipoxygenase (LOX) genes and activating their transcriptions. This study suggests MaWRKY70 plays a positive role in chilling injury development in banana fruit [contribution 15]. 

This editorial has summarized all research included in this Special Issue. The authors investigate the biologic and chemical changes underlying ripening and senescence, as well as the postharvest technologies of fresh, postharvest products for quality maintenance during storge time. Advanced physical approaches, including rapid cooling and CA packaging; chemical methods, such as ε-PL and chitosan coating, CMC-Gel-MT coating, pullulan coating, triterpenoids, and CaCl_2_; and biocontrol treatment technologies are recorded in this Special Issue. Thus, this Special Issue expands our knowledge of nutrition changes and molecular mechanisms and contributes advanced technologies for maintaining the shelf life and commercial value of fresh fruit and vegetables.

## References

[1] Guo Y., Ren G., Zhang K., Li Z., Miao Y., Guo H. (2021). Leaf senescence: Progression, regulation, and application. Mol. Hortic..

[2] Seymour G.B., Østergaard L., Chapman N.H., Knapp S., Martin C. (2013). Fruit development and ripening. Annu. Rev. Plant Biol..

[3] Figueroa C.R., Jiang C.Z., Torres C.A., Fortes A.M., Alkan N. (2021). Editorial: Regulation of Fruit Ripening and Senescence. Front. Plant Sci..

[4] Gambhir P., Raghuvanshi U., Kumar R., Sharma A.K. (2024). Transcriptional regulation of tomato fruit ripening. Physiol. Mol. Biol. Plants.

[5] Liu T., Albornoz K., Deltsidis A., Beckles D.M. (2022). Editorial: Postharvest ripening, senescence, and technology. Front. Genet..

[6] Wu J., Tang R., Fan K. (2023). Recent advances in postharvest technologies for reducing chilling injury symptoms of fruits and vegetables: A review. Food Chem. X.

[7] Habibi F., Boakye D.A., Chang Y., Casorzo G., Hallman L.M., Madison M., Clavijo-Herrera J., Sarkhosh A., Liu T. (2024). Molecular mechanisms underlying postharvest physiology and metabolism of fruit and vegetables through multi-omics technologies. Sci. Hortic..

[8] Feng Y., Xu Y., Li W., Chen S., Su Z., Xi L., Li G. (2022). Improved enrichment and analysis of heterocyclic aromatic amines in thermally processed foods by magnetic solid phase extraction combined with HPLC-MS/MS. Food Control.

